# Intraoperative Radiotherapy in the Management of Locally Recurrent Extremity Soft Tissue Sarcoma

**DOI:** 10.1155/2015/913565

**Published:** 2015-08-09

**Authors:** Christopher L. Tinkle, Vivian Weinberg, Steve E. Braunstein, Rosanna Wustrack, Andrew Horvai, Thierry Jahan, Richard J. O'Donnell, Alexander R. Gottschalk

**Affiliations:** ^1^Department of Radiation Oncology, University of California, San Francisco, 1600 Divisadero Street, MZ Building R H1031, San Francisco, CA 94143, USA; ^2^Department of Orthopaedic Surgery, University of California, San Francisco, San Francisco, CA, USA; ^3^Department of Pathology, University of California, San Francisco, San Francisco, CA, USA; ^4^Department of Medicine, University of California, San Francisco, San Francisco, CA, USA

## Abstract

*Purpose*. To investigate the efficacy and morbidity of limb-sparing surgery with intraoperative radiotherapy (IORT) for patients with locally recurrent extremity soft tissue sarcoma (ESTS).* Methods and Materials*. Twenty-six consecutively treated patients were identified in a single institution retrospective analysis of patients with locally recurrent ESTS treated with IORT following salvage limb-sparing resection from May 2000 to July 2011. Fifteen (58%) patients received external beam radiotherapy (EBRT) prior to recurrence (median dose 63 Gy), while 11 (42%) patients received EBRT following IORT (median dose 52 Gy). The Kaplan-Meier product limit method was used to estimate disease control and survival and subsets were compared using a log rank statistic, Cox's regression model was used to determine independent predictors of disease outcome, and toxicity was reported according to CTCAE v4.0 guidelines.* Results*. With a median duration of follow-up from surgery and IORT of 34.9 months (range: 4 to 139 mos.), 10 patients developed a local recurrence with 4 subsequently undergoing amputation. The 5-year estimate for local control (LC) was 58% (95% CI: 36–75%), for amputation-free was 81% (95% CI: 57–93%), for metastasis-free control (MFC) was 56% (95% CI: 31–75%), for disease-free survival (DFS) was 35% (95% CI: 17–54%), and for overall survival (OS) was 50% (95% CI: 24–71%). Prior EBRT did not appear to influence disease control (LC, *p* = 0.74; MFC, *p* = 0.66) or survival (DFS, *p* = 0.16; OS, *p* = 0.58). Grade 3 or higher acute and late toxicities were reported for 6 (23%) and 8 (31%) patients, respectively. The frequency of both acute and late grade 3 or higher toxicities occurred equally between patients who received EBRT prior to or after IORT.* Conclusions*. IORT in combination with oncologic resection of recurrent ESTS yields good rates of local control and limb-salvage with acceptable morbidity. Within the limitations of small subsets, these data suggest that prior EBRT does not significantly influence disease control or toxicity.

## 1. Introduction

With the demonstration of comparable overall and disease-free survival rates with oncologic resection coupled with adjuvant radiotherapy compared to amputation, limb-sparing surgery and radiation have become the standard curative therapy for extremity soft tissue sarcoma (ESTS) [1]. This multimodality approach yields long-term overall local failure rates of approximately 20% [1–3]. However, subsets of patient defined by clinicopathologic features, including surgical margins status and tumor grade, size, depth, and location, have varying risk of recurrence. For example, in carefully selected patients with small tumors resected and widely clear margins, a 10-year local recurrence rate of ~10% has been observed following surgery alone [4], while series of patients with positive surgical margins treated with adjuvant radiotherapy suggest ~30% local recurrence rate [5, 6]. For patients who experience an isolated local recurrence after definitive limb-sparing therapy, outcomes of both subsequent local control and overall survival are generally inferior to that observed with primary, localized disease, and yet it has long been recognized that a significant fraction of these patients can be salvaged [7–9].

The treatment algorithm of locally recurrent ESTS has been proposed to follow a similar workflow of that of primary ESTS, with long-term salvage local control rates ranging widely from 42 to 67% [10–12]. Data are conflicting regarding the role of adjuvant reirradiation in those patients treated initially with conservative surgery and radiation, however, with reports of superior local control through the use of combined surgery and reirradiation compared to surgery alone [13], while others have observed no significant improvement in local control with the addition of reirradiation [11]. Furthermore, significant postsalvage toxicity has been reported in patients treated with reirradiation [11, 13, 14]. While brachytherapy and external beam radiotherapy (EBRT) have historically been employed in the recurrent setting, more recently an alternative technique through the use of intraoperative external beam radiotherapy (IORT) has also been reported [15–18]. IORT may be used to rapidly deliver highly localized, high dose treatment with the added advantages of direct visualization of the tumor bed at the time of surgery, the ability to displace or shield nearby critical structures, and the possibility to administer lower dose adjuvant EBRT. With the exception of the recent large Spanish cooperative study [19], however, many of these reports have included analysis of patients with both primary and locally recurrent disease, making it more difficult to assess the role of IORT in the management of recurrent disease.

Since 1998 our institution has employed single fraction electron-based IORT through the use of a mobile linear accelerator for patients at risk for close or microscopically positive margins, in both the primary and recurrent setting, as well as for those patients with recurrent tumors within a previously irradiated field. In this study, we report mature treatment outcomes and toxicities for patients treated with limb-sparing surgery and IORT for locally recurrent ESTS at high risk for subsequent local recurrence.

## 2. Methods and Materials

### 2.1. Patients

The committee on human research approved this retrospective study of patients with extremity STS treated with IORT between May, 2000, and July, 2011, at the University of California, San Francisco (UCSF). Twenty-six consecutively treated patients were identified with a diagnosis of locally recurrent ESTS, with or without distant metastasis, who underwent limb-sparing reresection and IORT. STS were restricted to tumors within the “Soft Tissue Sarcoma” section of the 7th edition American Joint Committee on Cancer (AJCC) Staging Manual [20]. Workup included a history and physical exam, routine laboratory studies, and, depending on the individual case, exam under anesthesia, chest radiograph, computed tomography (CT) of chest, abdomen, and pelvis, magnetic resonance imaging (MRI) of the extremity of interest, or positron emission tomography (PET). Fine needle aspirate (FNA) or incisional biopsy was done prior to planned oncologic resections. Final tumor size was determined from pathologic data and AJCC staging using the retreatment classification was done at the time of reresection and IORT. The time to development of any initial relapse (local or distant) was defined from end of initial prerelapse treatment to first recurrence. Patients were presented and multimodality management recommendations made at the multidisciplinary UCSF sarcoma tumor board.

### 2.2. Surgery

A single senior orthopaedic oncologist (RJO) performed all definitive reresections. Pathology for all cases was reviewed at UCSF and assigned a grade according to the FNCLCC grading system (Coindre system) [21], with grade 1 tumors considered as low grade and grades 2 and 3 tumors considered as high grade. Surgical margins were considered positive when tumor cells were found at the margin of the resected specimen and close when margins were ≤2 mm. For patients where intraoperative margin status based on frozen section was reported (*n* = 20), there were no discrepancies on final permanent pathology.

### 2.3. Radiation

The indications for IORT and the target fields were determined jointly by the surgeon and radiation oncologist. The most common indications were the expectation of a close or positive margin adjacent to a critical structure and recurrence after prior irradiation. Additional indications included high grade pathology, bulky recurrence (>5 cm), and multiply recurrent disease. The Mobetron linear accelerator (IntraOp Medical Corporation, Nevada) was used at the time of definitive resection to deliver 4 to 12 MeV electrons through flat or beveled cones ranging from 2.5 to 10 cm in internal diameter. Dose was typically prescribed to the 85–90% isodose line. Total delivered dose was determined based on surgical bed volumes and intraoperative frozen pathologic margin status. Critical structures, particularly uninvolved nerves or vessels, were either mobilized away from the treatment field or protected with lead shielding. Tissue bolus was used in select cases to spare sensitive structures located deep to the tumor bed. Recommendations for adjuvant EBRT were based on the absence of prior in-field EBRT, final pathologic margins, and high risk disease (positive margins outside direct IORT field and bulky, deep, high grade disease). EBRT consisted of 3D conformal technique with a clinical target volume (CTV) encompassing the surgical bed, drain sites, and scar plus a 3 cm radial margin and a 5 cm longitudinal margin based on the preoperative gross tumor volume (GTV) extent defined on preoperative CT and/or MRI and was employed 4–8 weeks postoperatively.

### 2.4. Chemotherapy

Initiation of chemotherapy was based on high risk disease features (high grade disease, advanced group stage, and multiply recurrent tumors), symptomatology (pain and/or neuropathy), tumor proximity to critical structures, and desired presurgical shrinkage, as well as patient age, comorbidities, and performance status. The most commonly employed chemotherapy regimen consisted of a combination of anthracycline and ifosfamide.

### 2.5. Follow-Up

Patients were evaluated 3 to 6 weeks after reresection and IORT, then at 3- to 6-month intervals for disease status and toxicity assessments for 2 to 3 years, and then at annual intervals. Surveillance imaging of the site of interest and chest was obtained at 6- to 12-month intervals. Postreresection and IORT failure was defined at the time of biopsy or resection proven recurrence, except in cases of patient or physician deferred biopsy which were diagnosed clinically. Local recurrence was defined as a recurrence within the IORT and/or EBRT treatment field, while distant metastasis was defined as spread of the primary disease outside the irradiated field.

Local and distant metastasis-free disease control were each measured from the date of reresection and IORT to the date of recurrence, while the amputation-free duration was from the date of surgery and IORT to the date of amputation. The durations for patients without documented disease recurrence or amputation were censored at the date of last disease follow-up. Overall survival (OS) was defined as the period of time from the date of reresection and IORT to death from any cause. The durations for patients without documented death were censored at the date the patients were last known to be alive. Disease-free survival (DFS) was defined as the period of time from reresection and IORT to the date of first documented evidence of disease recurrence or death from any cause, whichever occurred first. The durations for surviving patients remaining disease-free were censored at the last date of follow-up. The time to initial recurrence was defined as the interval from end of initial definitive therapy to date of first recurrence.

Physician reported morbidity was assessed according to the Common Toxicity Criteria for Adverse Events, version 4.0 (CTCAE V4.0) [22], with acute and late events defined as those arising within 90 days or beyond 90 days of reresection and IORT, respectively. Recorded events were of those considered to be medically significant to severe (grade 3 or higher). To assess limb function, the worst individual toxicity was reported for joint function, weakness, or gait. Moderate wound complications included seromas requiring multiple aspirations and infections managed with operative debridement.

### 2.6. Statistical Analysis

Descriptive statistics (e.g., medians with minimum and maximum values and percentages) were calculated to summarize patient and disease features and treatment toxicities. Baseline and treatment subsets were compared using either Fisher's exact test for categorical features or *t*-test for continuous variables. The Kaplan-Meier product limit method was used to estimate the 5-year probabilities and presented with 95% confidence intervals (CIs) of overall survival and disease-free survival and of remaining free of local recurrence, metastatic recurrence, and amputation. Patient subsets were compared using a log rank statistic. Cox's regression model was used univariately to determine whether the duration to first recurrence was a predictor of each disease outcome following reresection and IORT. Significance was determined by the likelihood ratio (LLR) test with results summarized with a hazard ratio (HR) and 95% CIs. The same method was applied to identify independent predictors of each disease outcome. Due to the total sample size, following convention at most 2 predictors were included in a model. For all analyses a probability value less than 0.05 was considered to be statistically significant. Analyses were performed using Statistica (StatSoft v6).

## 3. Results

### 3.1. Patient Characteristics

The median age at the time of definitive reresection and IORT was 51 years with a range of 12 to 76 years. Upper extremity tumors were somewhat more common, occurring in 15 (58%) patients. Tumors were deep to the superficial fascia in 22 (85%) patients and 14 (54%) patients had tumors >5 cm in largest diameter. High grade (FNCLCC grade 2 or 3) tumors comprised the majority of cases (77%), with undifferentiated pleomorphic sarcoma (27%) and synovial sarcoma (19%) representing the most common histologies. AJCC group stage II or higher was found in 20 (77%) patients. Of the 6 patients with low grade tumors, 4 had positive margins, 5 were deep in location, and 4 were larger than 5 cm. A single local recurrence prior to reresection and IORT was observed in 20 (77%) patients, while 6 (23%) patients experienced more than one recurrence (range: 2–5). The median time to first local recurrence from the end of initial treatment was 17 months, with a range of 3 to 199 months. Close to half of first recurrences were detected beyond two years from initial treatment (46%). Distant metastasis prior to reresection and IORT was detected in 4 (15%) patients, all with spread to lung. The baseline patient characteristics are summarized in Table 1.

### 3.2. Treatment Characteristics

All patients achieved a gross total resection, yet margins were found to be microscopically positive in 12 (46%) specimens and close (≤2 mm) in 7 (27%). Median cone size used for IORT was 7 cm (range: 3–10 cm) and 5 (19%) patients were treated with >1 IORT field. Median IORT prescription dose was 15 Gy (range: 10–18 Gy). With the exception of one patient who received both EBRT prior to recurrence (60 Gy) and following reresection and IORT (40 Gy) and one patient who received only IORT, each patient who received EBRT prior to recurrence underwent IORT alone and each patient without a history of prior EBRT underwent both IORT and adjuvant EBRT. Median EBRT prescription dose prior to recurrence was 63 Gy with a range of 25 to 72 Gy. Median adjuvant EBRT following IORT was 52 Gy with a range of 22 to 60 Gy. Chemotherapy as part of initial definitive treatment prior to recurrence was given to 6 (25%) patients, while peri-IORT chemotherapy was given to 13 (54%) patients. A combination of anthracycline and ifosfamide containing regimen was the most commonly employed, and peri-IORT chemotherapy was restricted to high grade tumors in all cases. The treatment characteristics are summarized in Table 2.

### 3.3. Disease Control Outcomes

**Figure 1:**
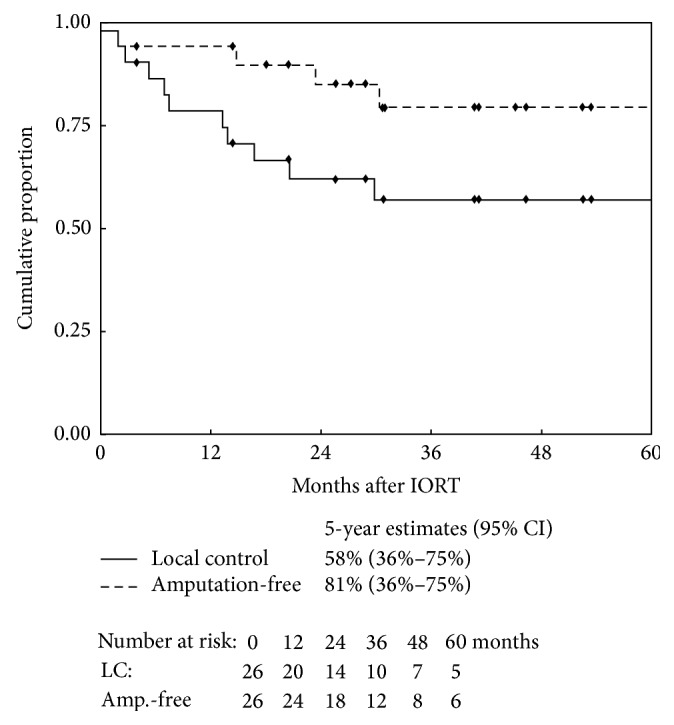
Kaplan-Meier probability distributions of local disease control and free of amputation after oncologic reresection and intraoperative radiotherapy (IORT) with 5-year estimates. CI = confidence interval.

**Figure 2:**
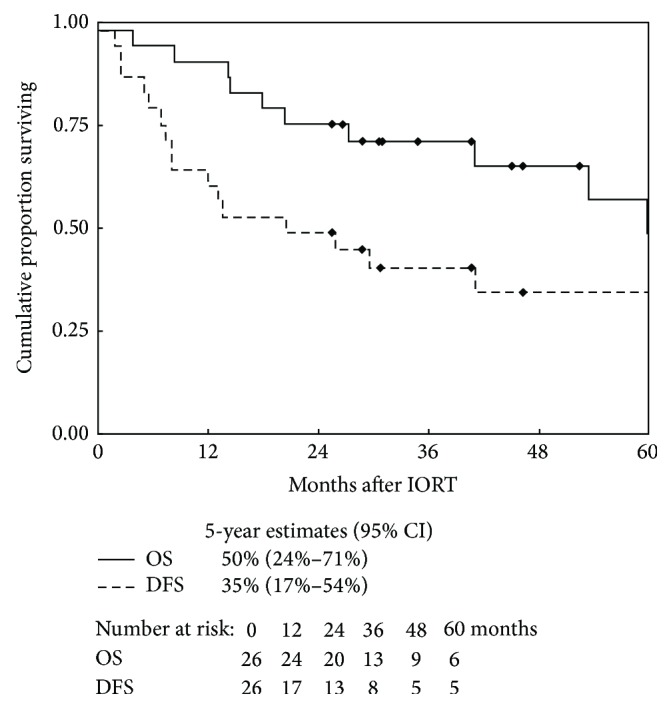
Kaplan-Meier probability distributions of disease-free survival (DFS) and overall survival (OS) after oncologic reresection and IORT with 5-year estimates. CI = confidence interval.

From the date of reresection and IORT, the median follow-up was 34.9 months (range: 4–139 months) for all patients and 45.1 months (range: 26–139 months) for living patients. Ten patients developed a local recurrence following reresection and IORT with a median duration of 10 months (2–30 months), and 10 patients developed new or progressive distant metastasis with a median duration of 8 months (2–50 months). Four patients with local recurrence subsequently underwent amputation. Of the 8 patients who experienced a local recurrence and were without metastasis at the time of reresection and IORT, 3 patients also developed distant metastasis, 2 at the time of local recurrence and 1 following local recurrence. Of the 4 patients with metastasis at the time of reresection and IORT, 2 developed a subsequent local recurrence and all four developed new or progressive distant metastasis. Of the 10 patients who have died, 9 expired with metastatic disease after disease recurrence, while one patient died without disease recurrence. The 5-year Kaplan-Meier estimate for local control was 58% (95% CI: 36–75%), for amputation-free was 81% (95% CI: 57–93%), for metastasis-free control was 56% (95% CI: 31–75%), for disease-free survival was 35% (95% CI: 17–54%), and for overall survival was 50% (95% CI: 24–71%) (Figures 1 and 2).

In comparing patients with or without a history of prior EBRT for initial management, there was no significant difference in any of the clinicopathologic variables analyzed (Fisher's exact test, *p* > 0.05). Analysis of disease outcomes by course of EBRT revealed no difference between those patients who received EBRT as part of their initial management followed by reresection and IORT alone compared to those who underwent adjuvant EBRT following salvage surgery and IORT (Log rank, *p* > 0.05). The Kaplan-Meier 5-year estimate of local control of those patients who received prior EBRT and those patients who did not receive prior EBRT was 55% and 61%, respectively. Using Cox's regression model the longer the duration of the time to first recurrence was found to be a significant predictor of prolonging metastasis-free control, disease-free survival, and overall survival but not local control (LLR test, *p* = 0.002, 0.009, 0.001, and 0.09, resp.). The interval to first recurrence was also a significant predictor of metastasis-free control, disease-free survival, and overall survival when limited to those patients without metastases at the time of reresection and IORT (*p* = 0.03, 0.04, and 0.02, resp.). T stage was the only independent predictor of local control for the overall patient cohort (*p* = 0.02, HR = 5.35, 95% CI: 1.13–25.4), while no significant predictor was found when limited to patients without metastasis at recurrence. The presence of metastasis at the time of reresection and IORT was a significant predictor of inferior overall survival (*p* = 0.04, HR = 5.70, 95% CI: 1.12–29.15).

### 3.4. Toxicity

Acute medically significant toxicity (grade 3) developed in 6 (23%) patients and late grade 3 toxicity developed in 8 (31%) patients, while no patient developed grade 4 or higher acute toxicity (Table 3). A total of 8 patients experienced any grade 3 toxicity with 7 developing multiple severe events. Gait disturbance and functional impairment of the limb due to joint stiffness or weakness were the most common events and occurred acutely in 3 (12%) patients and in the late phase in 6 (23%) patients, including the 3 patients with acute events. However, eventual improvement or resolution was noted in 5 of these patients. Significant toxicity related to limb/joint function occurred in 3 patients who received EBRT prior to recurrence and in 3 patients who received adjuvant EBRT following reresection and IORT. Acute grade 3 wound complications, both infectious and noninfectious, occurred in 3 (12%) patients, with 2 patients treated with EBRT prior to recurrence and 1 patient treated with EBRT after recurrence. Late grade 3 wound complications occurred in 4 (15%) patients including 2 who had acute events, with 3 patients treated with EBRT prior to recurrence and 1 patient treated with EBRT following recurrence. Each wound complication resolved with intravenous antibiotics and/or surgical intervention. Three patients experienced both wound and joint/limb function-related grade 3 toxicity.

## 4. Discussion

The value of adjuvant radiotherapy (RT) for improved local control following oncologic limb-preserving surgery of ESTS has been well established [1, 2, 23, 24]. In addition to several patient- and tumor-related predictive factors related to local control following this treatment paradigm, locally recurrent disease course has been associated with inferior local control [5, 7, 25]. When feasible salvage limb-sparing surgery remains the primary treatment modality for these patients [12], however, the role of radiation, particularly in the reirradiation setting, is less well defined [13, 14]. Given the potential for compounded toxicity in the recurrent setting, varied advanced radiation techniques including brachytherapy and IORT have been employed to deliver conformal high dose irradiation while minimizing normal tissue toxicity. The direct visualization provided by intraoperative external beam may have further advantages for targeting and critical structure protection and may allow for relatively reduced doses of large field adjuvant EBRT [26]. In this study, disease control and treatment toxicity outcomes were analyzed for patients treated at our institution for locally recurrent high risk ESTS treated with salvage limb-sparing resection and IORT.

Using an approach of salvage oncologic resection combined with IORT where adjuvant EBRT is largely restricted to those without prior radiation, we find encouraging long-term disease outcomes in this high risk population with 5-year local control, limb salvage, and overall survival estimates of 58%, 81%, and 50%, respectively. A limited number of groups have reported disease outcomes in cohorts of patients with locally recurrent ESTS treated with salvage limb-sparing surgery and IORT [15–19, 26–28]. While the majority of these studies have included patients with both primary and locally recurrent disease, with inclusion of patients with STS of various sites in some series, our results do appear to compare favorably with these reports. In the report from the group at the University of Heidelberg of 153 patients with ESTS, 58 patients with recurrent disease were included, the majority of whom (49 patients) received prior EBRT and underwent reresection and IORT alone for recurrence [26]. Interestingly, there did not appear to be a significant difference in outcomes between patients with recurrent and primary disease in this study, with 5-year estimates of local recurrence-free survival and OS of 69% versus 73% and 64% versus 78%, respectively. However, significant differences in patient and tumor characteristics or follow-up duration, if any, were not reported. A report from Stanford University of 50 patients with either locally advanced or recurrent STS of various sites (4 patients with ESTS) included 35 patients with recurrent disease, approximately half of whom (16 patients) received prior EBRT. Additional therapy, RT and/or systemic therapy, was given to 16 patients, and while the use of adjuvant EBRT appeared to significantly improve disease specific survival, the use of and outcomes with adjuvant EBRT in previously irradiated patients were not described. Overall 5-year patient outcomes were 55%, 24%, and 30% for in-field control (within the IORT field), locoregional control (within anatomic site), and disease-specific survival, respectively.

In a more recent report focusing on an extensive patient cohort with nonmetastatic recurrent STS, Calvo et al. [19] conducted a pooled analysis of 103 patients following reresection and IORT with or without adjuvant EBRT from three Spanish institutions with a median follow-up of 57 months. Approximately one-third of patients (*n* = 31) received EBRT prior to recurrence, 17 of whom underwent reirradiation. Two-thirds of the patients had extremity or trunk wall STS and one-third had retroperitoneal STS. Overall 5-year local control and disease-free survival estimates were 60% and 52%, respectively. Importantly, the use of adjuvant EBRT was a significant predictor of local control on multivariable analysis. While there did not appear to be a significant difference in local control (or other outcomes) between those patients with a history of prior EBRT and those who received reirradiation compared to all others, outcomes between those treated with reresection and IORT alone after failing EBRT and those treated without prior EBRT with reresection, IORT, and adjuvant EBRT were not separately reported. Collectively, disease control and survival outcome results from our cohort as well as those from the series described above are in line with those observed in patients with locally recurrent STS treated with reresection and either EBRT [11] or brachytherapy [29, 30].

With regard to toxicity, we found a moderate occurrence of both acute and late toxicities within this group of heavily treated patients, the majority of whom completed several rounds of multimodality treatment. Acute and late grade 3 toxicity developed in 23% and 31% of patients, respectively, and were predominantly related to gait disturbance or functional impairment of the limb due to joint stiffness or weakness. No grade 4 or 5 toxicity was observed, and no patient required amputation secondary to treatment associated toxicity. Toxicity reported from the Spanish cooperative study by Calvo et al. [19], assessed through the Radiation Therapy Oncology Group/European Organization for Research and Treatment of Cancer (RTOG/EORTC) criteria [31], included 16 (16%) patients with acute grade 3 or higher toxicity, mostly related to wound complications, while significant late toxicity developed in 13 (13%) patients and consisted predominantly of neuropathy and edema. While acute or late toxicity did not appear to differ significantly between those who received adjuvant EBRT and those that did not, toxicity related to reirradiation was not reported. Tran et al. [17] reported grades 3/4 toxicity in 4 (8%) patients, and this consisted of wound breakdown, fistula, neuropathy, and hydronephrosis. Acute grades 2–4 toxicity reported from the University of Heidelberg group developed in 35 (23%) patients, while late grades 2–4 toxicity, per RTOG/EORTC criteria, was reported in 26 (17%) of patients. Acute toxicity consisted predominately of wound-healing disturbances, while late toxicity was most commonly related to joint contracture/fibrosis and neuropathy. Evaluation of toxicity by disease course or prior EBRT was not specified in the above two studies. Overall, review of these data suggests that the moderate toxicity observed in this study is representative of those observed in patients with recurrent STS managed with salvage surgery and IORT. Furthermore, evaluation of toxicity from studies employing EBRT [11, 13, 14] or brachytherapy [29, 30] in the recurrent setting indicates that complications reported from our study are comparable, with significant complications ranging from 18 to 80%.

An interesting report, in abstract form, from members of the Spanish cooperative IORT group in which a similar treatment paradigm to that of ours for recurrent ESTS was employed, has suggested inferior local control and increased toxicity in patients who received prior EBRT [32]. Fifty patients with an isolated local recurrence of ESTS underwent reresection with either IORT or high dose-rate (HDR) brachytherapy. Adjuvant EBRT was given only to patients without prior local radiation (24 patients), while previously irradiated patients underwent reresection and IORT alone (26 patients). The five-year locoregional control estimate in patients with prior EBRT was significantly lower compared to those without prior EBRT (26% versus 81%, *p* = 0.001), and of the 13 (26%) patients with grade 3 or 4 toxicity, 9 patients required reintervention and all were within the prior EBRT group. In our series we did not find statistically significant differences in either disease control outcomes or toxicity between previously irradiated patients and those without prior EBRT. Reasons for these discrepancies are unclear, yet may reflect differences in our highly selected study populations, IORT application (electron beam versus brachytherapy), or other unforeseen variables. It must also be acknowledged that limitations to our study, as well as many of the described studies above, including the retrospective study design and accompanying small sample size limit definitive conclusions are at best hypothesis generating. An additional possible limitation to this study is the inclusion of patients with known distant metastatic disease at the time of recurrence. Indeed, each of the four patients with metastatic disease at the time of initial recurrence progressed distantly and succumbed to their disease.

In conclusion, this study demonstrates that incorporation of IORT as a component of multimodality management of patients with locally recurrent ESTS achieves high rates of local control and limb salvage with acceptable treatment morbidity and suggests that prior EBRT does not significantly influence disease control or toxicity. This technique offers a method of delivering focal therapy to achieve local control for the majority of patients who otherwise may require more radical surgical procedures. Larger, prospective trials are necessary to more fully evaluate the role of IORT in disease control and toxicity outcomes in patients with ESTS.

## Figures and Tables

**Table 1:** Baseline patient characteristics at time of reresection and IORT (*n* = 26).

Patient characteristic	Number of patients (%)
Age	
Median	51 years
Range	12–76 years
≤18	1 (4%)
19–50	12 (46%)
>50	13 (50%)

Gender	
Female	15 (58%)
Male	11 (42%)

Site	
Lower extremity	11 (42%)
Upper extremity	15 (58%)

Histology	
Undifferentiated pleomorphic sarcoma	7 (27%)
Synovial sarcoma	5 (19%)
Liposarcoma	4 (15%)
Fibrosarcoma	3 (11.5%)
Spindle cell sarcoma	3 (11.5%)
Leiomyosarcoma	1 (4%)
Malignant peripheral nerve sheath tumor	1 (4%)
Rhabdomyosarcoma	1 (4%)
Soft tissue sarcoma, NOS	1 (4%)

T stage	
T1	12 (46%)
T2	14 (54%)

Depth	
Superficial	4 (15%)
Deep	22 (85%)

Grade (FNCLCC)	
1	6 (23%)
2	6 (23%)
3	14 (54%)

AJCC group stage	
I	6 (23%)
II	11 (42%)
III	5 (19%)
IV	4 (15%)

Initial local recurrence type	
Single	20 (77%)
Multiple	6 (23%)

Time to initial recurrence	
Median	17 mo.
Range	3–199 mo.

**Table 2:** Treatment characteristics.

Treatment characteristic	Number of patients (%)
Surgical margins	
Positive	12 (46%)
Close (≤2 mm)	7 (27%)
Negative	7 (27%)

Radiation therapy	
EBRT prior to recurrence	15 (58%)
Median dose (range)	63 Gy (25–72)
EBRT following IORT	11 (42%)
Median dose (range)	52 Gy (22–60)
IORT cone size	
Median	7 cm
Range	3–10 cm
IORT dose	
Median	15 Gy
Range	10–18 Gy

Chemotherapy (*n* = 24)	
As part of initial therapy	6 (25%)
Peri-IORT	13 (54%)
Peri-IORT chemotherapy schedule	
Pre-IORT alone	6 (25%)
Post-IORT alone	6 (25%)
Pre- & Post-IORT	1 (4%)

**Table 3:** Incidence of grade 3 acute and late toxicity.

Toxicity	Acute^*∗*^ Number of patients (%)	Late^*∗*^ Number of patients (%)
Wound complications		
EBRT prior to recurrence	2 (8%)	3 (15%)
EBRT following IORT	1 (4%)	1 (4%)
Limb/joint dysfunction		
EBRT prior to recurrence	1 (4%)	3 (12%)
EBRT following IORT	2 (8%)	3 (12%)

Total	6 (23%)	8 (31%)

^*∗*^Five of the 6 patients with grade 3 acute toxicity also had late toxicity of the same type, and 7 patients had multiple grade 3 toxicities.
